# Data article on soil site suitability analysis using geostatistical and visualization techniques for selected winter crops in Sagar island, India

**DOI:** 10.1016/j.dib.2020.105930

**Published:** 2020-06-26

**Authors:** Satyabrata Mandal, Burhan U. Choudhury, Lakshminarayan Satpati

**Affiliations:** a Department of Geography, University of Calcutta, 35 BCE Road, Kolkata 700019, India; b Division of NRM, ICAR Research Complex for NEH Region, Umiam, Meghalaya-793103, India

**Keywords:** Soil suitability, Soil quality index, Remote sensing and gis, Landuse planning, Crop diversification, coastal Island

## Abstract

We assessed soil site suitability for selected winter crops in the coastal saline agro-ecological environment of Sagar Island, India by integrating land limitation and crop suitability evaluation framework of FAO. Grid based (1 km by 1 km) soil sampling and estimation of important soil quality attributes were measured in the laboratory following standard procedures. Geo-statistical and visualization methods were applied to match the soil suitability for selected crops. The weights of crop specific soil parameters have been assigned through PCA analysis. The inverse distance weighting interpolation and reclassification methods were adopted for generation of spatial layers of those soil attributes. Nearly 61% area (14,618 ha GA) of the Island is under agricultural landuse (AL), mostly dominated (>75% of AL) by lowland rice-fallow mono-cropping. Soils are highly suitable (S1) for growing sunflower while moderately suitable (S2) for growing chilli, mustard and potato crops. The grid-wise georeferenced soil data information generated in this study will help in periodic monitoring of soil quality in spatio-temporal dimensions for devising location specific soil health managements in the Island. The methodology used in estimating soil quality index and crop specific soil suitability analysis in spatial format will help in replicating such studies in other such coastal Islands of Indian Sub-continent.

Specifications table

**Table:** 

Subject	Agricultural and Biological Sciences
Specific subject area	Soil Science
Type of data	Chart (Excel sheet)
How data were acquired	The topographical maps (79C/1, 79C/2; RF 1:50,000), Geological quadrangle maps (RF 1:25,000) and Block maps (RF 1:150,000) of Sagar Island collected from Survey of India (SOI), Geological Survey of India (GSI) and National Atlas and Thematic Mapping Organization (NATMO), Kolkata, respectively were digitized and converted into Google earth compatible format (.kml) for over-lay analysis in ArcGIS. High resolution (<1 m) Google satellite images were mosaicked into a single image, clipped with Island boundary and onscreen digitization was performed to prepare LULC map of the Island. From the LULC map, agricultural area (14,618 ha) was demarcated and divided into 251 grids (at 1 km by 1 km) to cover the Island. Soil survey and grid based geo-referenced (using GARMIN *etrex*10 GPS device) composite (of 3) soil samples from each of 251 grids were collected.
Data format	Raw and analysed data
Parameters for data collection	Pre-monsoon dry agricultural fellow period (April 2017) was considered for collection of grid based (1 km by 1 km within agricultural area derived from LULC) geo-referenced composite (of 3) samples from each grid cell. We took precautionary measures during sample collection so that we can get samples in usable condition during laboratory tests for soil quality properties of particle size distribution (sand, silt, clay) texture, pH, organic carbon; SOC, electrical conductivity; EC and available macronutrients- NPK.
Description of data collection	From each grid cell, 3 geo-referenced surface (0–15 cm) soils to make one composite sample were collected following standard protocol of sampling collection. Grid based (1 km by 1 km) collected soil samples were analysed in laboratory following standard procedures. Thematic maps were generated using IDW interpolation and reclassification methods in GIS. Soil quality index was estimated and geo-statistical and visualization methods were applied to match the suitability for selected crops.
Data source location	Sagar Island in the continental shelf of Bay of Bengal, located at the extreme southern end of Sundarbans Region, West Bengal, India (21° 37′ 20″ N to 21° 52′ 28″ N latitude and 88° 2′ 17″ E to 88° 10′ 25″ E longitude, with an elevation range of 2.5–3.5 m above mean sea level).
Data accessibility	Data are available with the article
Related research article	Authors name Satyabrata Mandal, Burhan U. Choudhury, Lakshminarayan Satpati Title Soil site suitability analysis using geo-statistical and visualization techniques for selected winter crops in Sagar Island, India Journal Applied Geography DOI https://doi.org/10.1016/j.apgeog.2020.102249

## Value of the data

• The high resolution datasets prepared will help in periodic monitoring of spatio-temporal changes in soil quality attributes and location specific soil health management.
• The finer resolution landuse map prepared will aid in assessing temporal changes in landuse pattern and accordingly, in devising sustainable landuse plan for this fragile Island.
• Farmers of the Island, policy makers for disaster management and sustainable landuse planning, researchers from wide range of subject areas like geographers, agronomist and environmentalists in and around similar coastal environment may be benefited with this data.
• This finer resolution spatial dataset generated through extensive ground survey, laboratory analysis and geo-spatial thematic products will act as benchmark dataset for further insights/experiments on soil suitability dimensions of other major crops.
• Dataset bears the opportunity to directly apply by the concerned researchers/environmental scientists in exploring multi-dimensional management aspects while forgoing huge labour, time and financial investments for acquiring the generated information.

## 1 Data

**Fig. 1:**
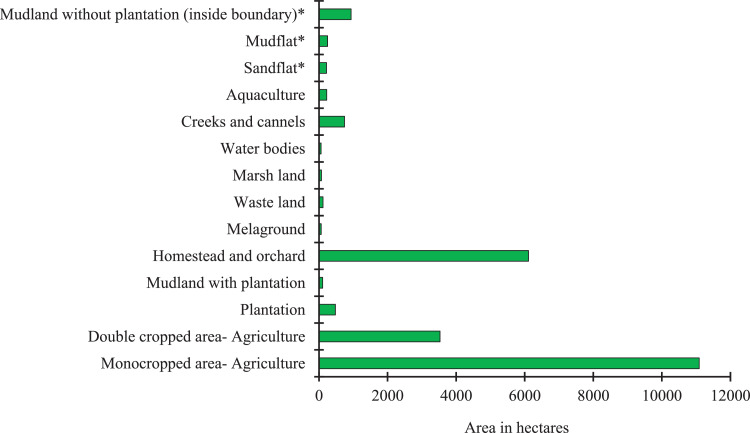
Area under major landuse and land cover category in Sagar Island, India. *:Sandflat/ mudflat/ mudland area varies frequently since they are affected by tidal gushes, flooding and coastal erosion on regular baisis.

**Fig. 2:**
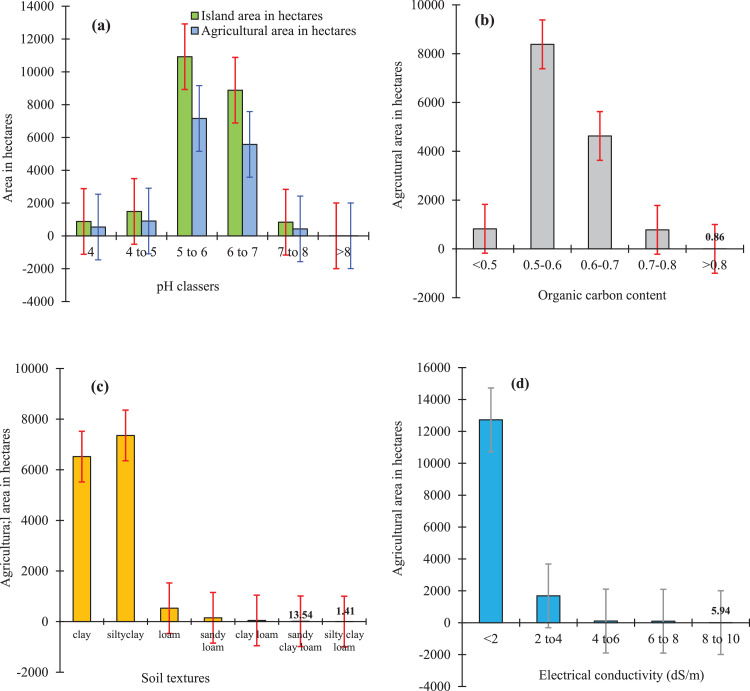
Agricultural area (hectares) under different classes of (a) soil pH, (b) Soil Organic Carbon (SOC), (c) Soil texture, (d) and Electrical Conductivity (EC) in Sagar Island, India.

**Fig. 3:**
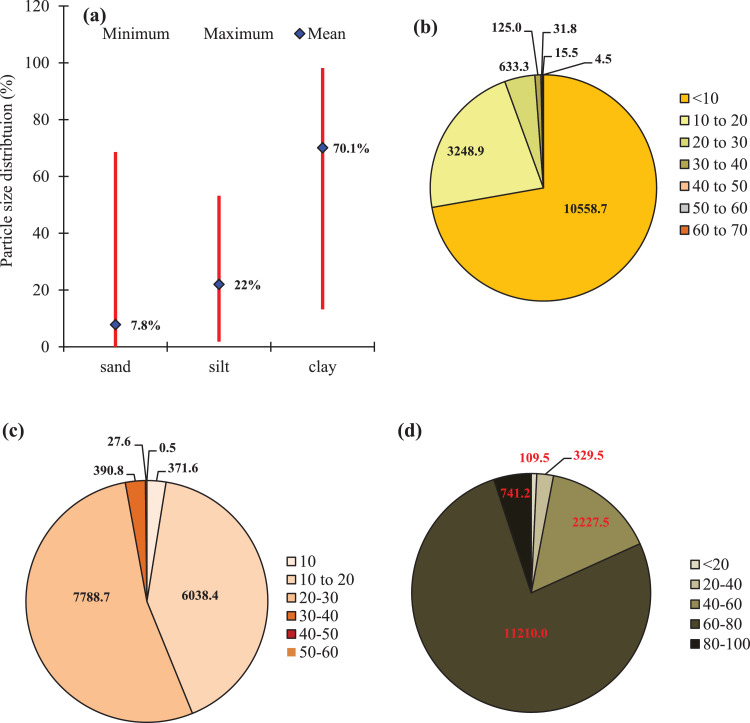
(a) Particle size distribution (sand, silt and clay in percent) in the soils of Sagar Island, (b) distribution of different proportions of sand, (c) silt and (d) clay (in percent) within the agricultural area (14,618 ha) of the Island.

**Fig. 4:**
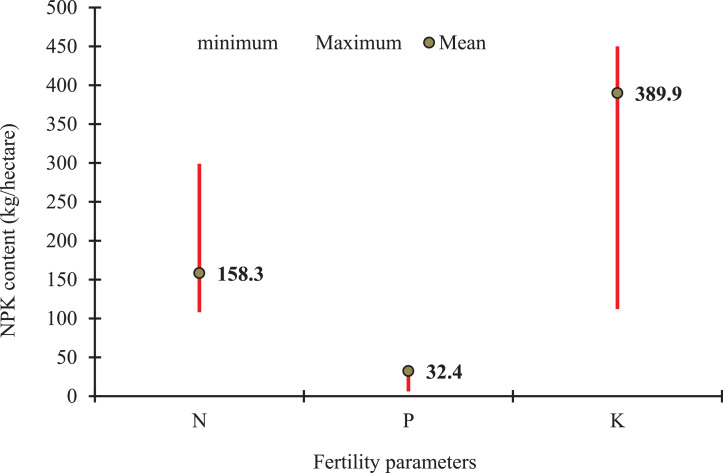
Fertility status (range and mean content of NPK) in the soil of Sagar Island.

**Fig. 5:**
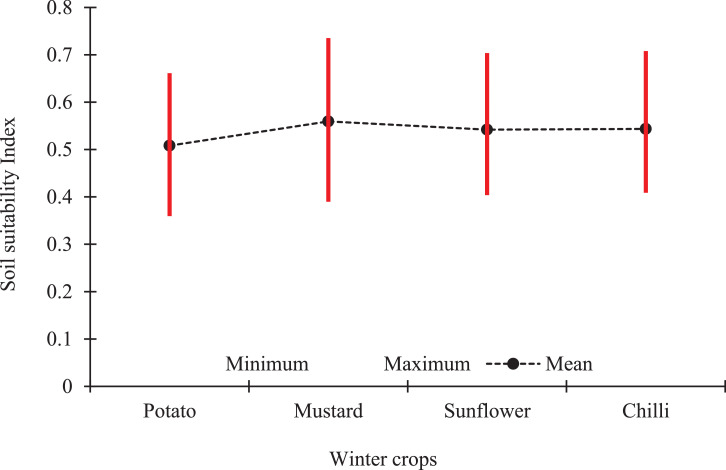
Mean and range of suitable indices of different winter crops in Sagar Island.

The data set contains tables and figures on landuse land cover pattern, distribution of soil properties, and the soil suitability analysis of winter crops (Sunflower, Chilli, potato and mustard) in the Sagar Island. Table 1 and Fig. 1 represent area and percent of total geographical area under different landuse land covers derived from satellite images. Tables 2 and 3 represent variation in soil properties and the estimated soil suitability index values for the winter crops in the Island. Fig. 2, Fig. 3, Fig. 4 represent areas under different classes and variation of soil properties (pH, SOC, EC, particle size distribution, macro-nutrients NPK percent distribution) while Fig. 5 represents variation in soil suitability index across the Island. We also supplied raw data on LULC classes (with pixel numbers), measured soil properties, classes of soil properties and in detail the methodology we adopted in estimation of soil quality indices for the four above mentioned different crops in excel sheets.

**Table 1:** Different types of Landuse and land cover (LULC) in Sagar Island, India.

Landuse type	Area in Hectares	Island area within boundary	% Island Area
Monocropped area- Agriculture	11,089.2	24,000	46.2
Double cropped area- Agriculture	3528.5	24,000	14.7
Plantation	477.4	24,000	2.0
Mudland with plantation	106.6	24,000	0.4
Homestead and orchard	6110.4	24,000	25.5
Melaground	65.1	24,000	0.3
Waste land	116.4	24,000	0.5
Marsh land	73.5	24,000	0.3
Water bodies	61.0	24,000	0.3
Creeks and cannels	744.5	24,000	3.1
Aquaculture	223.8	24,000	0.9
Sandflat*	218.2	24,000	0.9
Mudflat*	249.0	24,000	1.0
Mudland without plantation (including bounadry length)*	936.6	24,000	3.9
Total	24,000.0	24,000	100.0

**Table 2:** Descriptive statistics of soil properties in the soil of Sagar Island.

Descriptive statistics	Sand (%)	Silt (%)	Clay (%)	pH (1: 2 soil water ratio)	EC (1: 2 soil water ratio), dS/m	SOC (%)	Available N (kg/ha)	Available P (kg/ha)	Available K (kg/ha)
Minimum	0.00	1.8	13.2	4.25	0.190	0.41	108	6.11	112
Maximum	68.60	53.2	98.2	8.26	10.080	0.82	299	39.30	450
Mean	7.8	22.0	70.1	6.1	1.6	0.6	158.3	32.4	389.9
SD	11.93	8.67	14.79	1.00	1.65	0.12	55.60	7.97	82.23
CV (%)	152.0	39.3	21.1	16.4	101.0	21.1	35.1	24.6	21.1
sk	2.16	0.39	−0.99	0.09	2.89	0.38	0.79	−0.90	−1.29
ku	5.46	0.82	1.32	−1.12	9.48	−1.29	−0.94	−0.12	0.75

Soil particles measured in percent per unit volume of soil, pH and Electrical Conductivity measured in 1:2 soil water ratio, Fertility parameters of NPand K measured as kg per hectares land.

**Table 3:** Descriptive statistics for soil suitable indices of different winter crops in Sagar Island.

Descriptive statistics	Soil Suitability Index			
	Potato	Mustard	Sunflower	Chilli
Minimum	0.36	0.39	0.40	0.41
Maximum	0.66	0.74	0.70	0.71
Mean	0.51	0.56	0.54	0.54
SD	0.06	0.07	0.06	0.06
CV	11.59	11.72	11.54	11.39
Sk	0.13	0.15	0.13	0.12
Ku	−0.42	−0.35	−0.46	−0.47

## 2 Experimental design, materials, and methods

### 2 1. soil sampling and laboratory analysis

The study area was divided into 251 grid cells (1 km x 1 km) in order to ensure spatially uniform soil sampling. A total of 287 **c**omposite (of three samples) geo-referenced (through GARMIN *etrex*10 GPS device) surface (0–15 cm) soil samples were collected during pre-monsoon dry month of April 2017 from the agricultural fallow, after the harvest of predominant *kharif* rice on November/December 2016. A minimum of one composite sample from each of 251 grid cells with exception to few grids (36 numbers) having two composite samples were collected since they had relatively more areas under agricultural fallow (rice-fallow). So, all together, we collected 287 geo-referenced samples from 251 grid cells (1 km by 1 km) (see in Supplementary data) to represent the agricultural area of the Island. Kharif rice (*Aman*) is the predominant crop (cultivated in >75% of agricultural area) and rice-fallow is the predominant cropping system of the Island [1]. While collecting the soil samples, ancillary information (location, past cropping, management history and proposed crops etc.) were also recorded. Collected samples were air dried, ground and sieved to pass through 2-mm sieve and 0.5 mm for soil organic carbon (SOC). The processed soil samples were analysed for pH (1:2 soil: water) and EC (1:2 soil: water) following standard procedures [2]. The particle size distribution was determined by sieving for sand fractions while Robinson pipettes method for silt and clay fractions [3]. The soil texture was classified based on USDA textural classification scheme. The SOC content was determined from 0.5 mm sieved samples by the Walkley–Black method [4]. Available nitrogen was determined by alkaline potassium permanganate method following Subbaiah & Asija method [5]. Available Phosphorus was estimated with 0.5 M sodium bicarbonate (NaHCO3) (pH 8.5) proposed by [6] and a spectrophotometer was used to determine P content in the extract using ascorbic acid as reducing agent [7]. Available K was extracted with neutral 1 N ammonium acetate [8] and estimated by a flame photometer.

### 2.2 LULC mapping

The topographical maps (79C/1, 79C/2; RF 1:50,000), Geological quadrangle maps (RF 1:25,000) and Block maps (RF 1:150,000) of the study area were collected from the Survey of India (SOI), Geological Survey of India (GSI) and National Atlas and Thematic Mapping Organization (NATMO), Kolkata, respectively. All the maps were scanned and converted into digital form. The scanned topographical maps were geo-referenced using several ground control points (GCPs) of known latitude and longitude values in Arc GIS v10.2 software environment. Remaining maps were geo-referenced using image to image referencing technique. The digitized Island boundary (including mouza boundary) has been converted from Arc GIS default file format (.shp) to Google earth compatible format (.kml). The downloaded high resolution (<1 m) Google satellite images (February 10, 2012) were mosaicked into a single image and converted into projected coordinate system (northing/easting) using Universal Transverse Mercator (UTM) projection with standard procedures in GIS platform [9]. Finally, the image was clipped with Island boundary and onscreen digitization was performed to prepare LULC map of the Island.

### 2.3 Conceptual framework and criteria rating

The present study analysed the soil site suitability for selected winter crops (chilli, potato, sunflower and mustard) for Sagar Island using geo-statistical and visualization techniques in GIS and geo-statistical environment. The FAO frame work on land evaluation [10] modified by Naidu et al. [11] was adopted for soil suitability analysis. Seven important soil parameters i.e. soil texture; soil reaction (pH), organic carbon (SOC), electrical conductivity (EC) and available macronutrients- like nitrogen (N), phosphorus (P) and potash (K) were used in the suitability evaluation. The climatic parameters were considered as spatially homogeneous in nature because of the smaller spatial extensions of the Island and thereby, excluded from the analysis. Similarly, the soil slope (varied from 03%) and stoniness (nil) was considered as non-limiting factors of production hence, not considered. Based on the intensity of limitations, location specific soil suitability was classified as highly suitable (S1), moderately suitable (S2), marginally suitable (S3), not suitable (N) and permanently not suitable (N1) for each of the crops. The specific requirements of each crop (Table 1) was compared with the soil properties and based on the extent of matching, the areas under different levels of suitability was arrived.

### 2.4 Assign weights through principal component analysis (PCA)

The first principal component (PC1) of a set of variables has defined as the linear index that captured the largest amount of information common to all the variables [12].

Suppose the data matrix contained N-variables (*a_1j_ to a_Nj_*) that represents the N indicators of each sample point *j*. PCA expressed the indicators as linear combination of set of underlying components for each sample point *j*:

$$a_{1j}=\gamma_{11}A_{1j}{+}_{\gamma 12}A_{2j}+\ldots {+}_{\gamma 1N}A_{Nj;}(j=1\ldots J$$

(1) $$a_{Nj}=\gamma_{N1}A_{1j}+\gamma_{N2}A_{2j}+\ldots +\gamma_{NN}A_{Nj},$$

Where the A's are the components and the *γ’s* are the coefficients on each component for each variable. Technically, the pkrocedure solves the equation (*R –λnI) vn = 0 for λn and vn,* where *R* is the matrix of correlations between the scaled variables (*a*’s) and v*n* is the vector of coefficients on the *n*^th^ component for each variable. Solving the equation yields the characteristic roots of *R, λn* (also known as Eigen values), and their associated Eigen vectors, *vn.* The final set of estimates was produced by scaling the *vns,* so the sum of their squares sums to the total variance; this is another restriction imposed to achieve determinacy of the problem [13].

The model recovered scoring factors from inverting the system implied in Eq. (1). This yields a set of estimates for each of the A-PCs:

$$A_{1j}=f_{11}a_{1j}+f_{12}a_{2j}+\ldots +f_{1N}a_{Nj;\ldots .}\left(j=1\ldots J\right)$$

(2) $$A_{Nj}=f_{N1}a_{1j}+f_{N2}a_{2j}+\ldots +f_{NN}a_{Nj}$$

Where, the *f’*s are the factor scores.

Using the computed factor score matrix for each of the selected crops, the weighted scores (WSs) were assigned to the factors based on their relative importance on crop growth separately. All the seven soil parameters or variables were considered as indicators. Subsequently, a score was given to each indicator, and then the boundaries and shape of the scoring functions were set. This enabled in developing a composite index by normalizing and transforming units of all indicators into uniform scales (0–1) using 3 types of standardized scoring functions (i) more is better (ii) less is better, and (iii) optimum is better. Indicators were ranked in ascending or descending order depending on whether a higher value was considered ‘good’ or ‘bad’ in terms of soil function. For ‘more is better’ indicators, each observation was divided by the highest observed value (a score of 1). For ‘less is better’ indicators, the lowest observed value (in the numerator) was divided by each observation (in the denominator) such that the lowest observed value received a score of 1 [14]. Each individual variables of the seven soil parameters was assigned a weight, which was calculated as the ratio of the indicator factor loading to the cumulative component load or factor load of PCA analysis with Eigen value >1.0. Finally, the total scores (TSs) for each soil parameters for a specific crop has been computed by multiplying the PCA weights (as estimated in factor loadings) to the weighted scores (WSs) of corresponding locations.

Then the TSs of the parameters for all the selected crops were spatially interpolated using inverse distance weighting (IDW) method and those layers further reclassified in the Arc GIS v. 10.2 environment. The TSs for all the sample points for a specific crop was categorized into different classes such as highly suitable (S1), moderately suitable (S2), marginally suitable (S3) and not suitable (N). In Sagar Island, the TSs for chilli crop was categorized as <0.48 (S1), 0.48–0.58 (S2) and >0.58 (S3). Similarly, <0.50 (S1), 0.50–0.55 (S2) and >0.55 (S3) for potato crop; <0.50 (S1), 0.50–0.55 (S2) and >0.55 (S3) for sunflower crop while, this was <0.55 (S1), 0.55–0.65 (S2) and > 0.65 (S3) for mustard crop. Finally, suitability maps were generated by within agricultural area by masking off all the non agricultural attributes. Zonal geometry of these raster layers was extracted using spatial analyst tool. Apart from this, the thematic layers of all the soil parameters were generated using the geo-referenced laboratory test data. These raster surfaces were interpolated using IDW method and those layers were reclassified according to the crop requirements as mentioned in Table 1.

Finally, total score (TS) was estimated (more or less comparable to soil quality index) for different locations using the following formula [14].

$$Total\;score\;\left(TS\right)=\sum_{i=1}^{n}Wi\;Vi$$

Where, *Wi* = Weight of variables and *Vi* = Score of variables

The composite distribution of available macronutrients (NPK) class was spatially interpolated by their logical combinations. A total of 18 logical combinations were identified of which 7 combinations exhibited null pixel values, hence rejected. Therefore, the final NPK composite map was prepared with the 11 combinations/classes. Aside, Pearson's correlations matrix was used to evaluate the relationships between soil properties and available nutrients [15].

## Declaration of competing interest

We declare that we have no conflicts of interest.

## Appendix Supplementary materials

Image, application 1

Image, application 2
